# Effect of Surgical Procedures for Rheumatoid Forefoot Deformities on Radiographic Foot Length and Width Variations

**DOI:** 10.3390/jcm15051877

**Published:** 2026-02-28

**Authors:** Masahiro Horita, Yohei Kiso, Yoshihisa Nasu, Ryuichi Nakahara, Kenta Saiga, Toshifumi Ozaki, Keiichiro Nishida

**Affiliations:** 1 Department of Orthopaedic Surgery, Faculty of Medical Development Field, Okayama University, Okayama 700-8558, Japan; 2 Department of Orthopaedic Surgery, Kurashiki Sweet Hospital, Okayama 710-0016, Japan; yk.aprilfool@gmail.com; 3 Department of Orthopaedic Surgery, Okayama City Hospital, Okayama 700-8557, Japan; nasu_y@flute.ocn.ne.jp; 4 Department of Musculoskeletal Health Promotion, Faculty of Medicine, Dentistry and Pharmaceutical Sciences, Okayama University, Okayama 700-8558, Japan; pikumin55@gmail.com; 5 Department of Sports Medicine, Faculty of Medicine, Dentistry and Pharmaceutical Sciences, Okayama University, Okayama 700-8558, Japan; ksaiga7@gmail.com; 6 Department of Orthopaedic Surgery, Faculty of Medicine, Dentistry and Pharmaceutical Sciences, Okayama University, Okayama 700-8558, Japan; tozaki@md.okayama-u.ac.jp; 7 Locomotive Pain Center, Faculty of Medical Development Field, Okayama University, Okayama 700-8558, Japan; knishida@md.okayama-u.ac.jp

**Keywords:** forefoot surgery, foot length, foot width, rheumatoid arthritis

## Abstract

**Background**: The number of patients with rheumatoid arthritis (RA) undergoing forefoot arthroplasty has increased to better control the disease. Despite patients frequently expressing concerns regarding postoperative foot appearance and footwear-related expectations, no study has investigated postoperative changes in foot length and width in patients with RA. The aim of this study was to evaluate the effect of surgical procedures for rheumatoid forefoot deformities on variations in radiologically determined foot length and width. **Methods**: In total, 72 feet of 50 women and 3 men (average age: 66.7 years) underwent joint-preserving arthroplasty (*n* = 33) and arthrodesis of the first metatarsophalangeal joint with shortening osteotomy of the lesser metatarsals or resection arthroplasty of the lesser metatarsal heads (*n* = 39); procedures were carried out in our institute from August 2013 to February 2020. The mean disease duration was 23.5 years, and the average follow-up period was 17.5 months. Pre- and postoperative hallux valgus angle (HVA), intermetatarsal angle (IMA) of the first and second metatarsals (M1M2A), and IMA of the first and fifth metatarsals (M1M5A) were measured on weightbearing radiographs as well as foot length and width. We also evaluated the correlation between changes in radiographic parameters and variations in radiologically determined foot length and width. **Results**: Radiologically determined foot width changed significantly from 10.1 cm to 9.7 cm (*p* < 0.01), while no significant difference was found between pre- and postoperative radiologically determined foot length. HVA, M1M2A, and M1M5A were significantly improved after the surgery (*p* < 0.01, *p* < 0.01, and *p* < 0.01, respectively). A significant negative correlation was found between the variation in radiologically determined foot length and changes in HVA (r = −0.29, *p* = 0.02) and M1M5A (r = −0.23, *p* < 0.05), while a significant positive correlation was found between the variation in the foot width and changes in HVA (r = 0.34, *p* < 0.01), M1M2A (r = 0.55, *p* < 0.01), and M1M5A (r = 0.45, *p* < 0.01). There were no significant differences between operative procedures regarding variation in radiologically determined foot length and width. **Conclusions**: Surgical procedure for rheumatoid forefoot deformity improved radiographic parameters and reduced radiographic foot width while maintaining foot length.

## 1. Introduction

Feet are commonly an issue in patients with rheumatoid arthritis (RA). RA forefoot deformities include conditions such as hallux valgus (HV) and subluxation or dislocation of the lesser toes at the metatarsophalangeal (MTP) joints [1]. The presence of these foot alterations leads to increased forefoot width and decreased foot length [2,3,4]. The increase in foot width is often associated with difficulties with footwear [5]. Therefore, foot width reduction has been reported to be a desirable cosmetic and functional outcome for patients with HV [5]. Patients with RA may require joint surgery to improve their physical function and quality of life (QOL). This is partially due to their increased expectations [6]. According to a Japanese cohort study, the number of patients with RA undergoing foot arthroplasty has increased due to improved disease management [7]. Previous reports have suggested that patient satisfaction and QOL after HV surgery are more strongly influenced by foot appearance and the ability to wear preferred footwear than by either hallux valgus angle (HVA) or intermetatarsal angle (IMA) correction [8,9].

Despite patients frequently expressing concerns regarding postoperative foot appearance and footwear anticipations [9], no previous study has examined the effect of surgical procedures for forefoot deformities on foot length and width variations. The aim of this study was to evaluate the effect of surgical procedures for rheumatoid forefoot deformities on variations in radiologically determined foot length and width. We hypothesized that surgical procedures for rheumatoid forefoot deformities would result in significant decreases in radiologically determined foot width while maintaining foot length as measured on weightbearing radiographs.

## 2. Materials and Methods

This retrospective research was approved by the ethics committee of our university hospital (approval number: 2194). Seventy-two feet from 50 women and 3 men with RA underwent joint-preserving arthroplasty and arthrodesis of the first metatarsophalangeal joint with shortening osteotomy of the lesser metatarsals or resection arthroplasty of the lesser metatarsal heads for rheumatoid forefoot deformities in our university hospital from August 2013 to April 2020. All patients met the American Rheumatism Association’s 1987 revised criteria for RA and 2010 RA classification criteria [10,11]. The minimum follow-up period was 6 months after surgery. The mean disease duration was 23.7 ± 9.3 years and the mean follow-up period was 17.5 ± 10.4 months. The mean disease activity score 28 c-reactive protein (DAS28-CRP) [12] was 2.5 ± 0.8 and the mean health assessment questionnaire–disability index score [13] was 1.0 ± 0.7. Patient demographics are shown in Table 1.

The severity of the destruction of the MTP joint was assessed using the Larsen grade [14]. The operative indication was followed according to our previous reports [15,16]. Thirty-three feet from 25 women (mean age 64.7 ± 9.5 years) underwent corrective osteotomy of the first metatarsal bone with SOO of the lesser toe (joint-preservation group), while thirty-nine feet from 26 women and 3 men (mean age 67.8 ± 7.4 years) underwent arthrodesis of the first MTP joint with shortening osteotomy of the lesser metatarsals or resection arthroplasty of the lesser toe (joint-sacrifice group).

### 2.1. Radiographic Assessment

**Figure 1:**
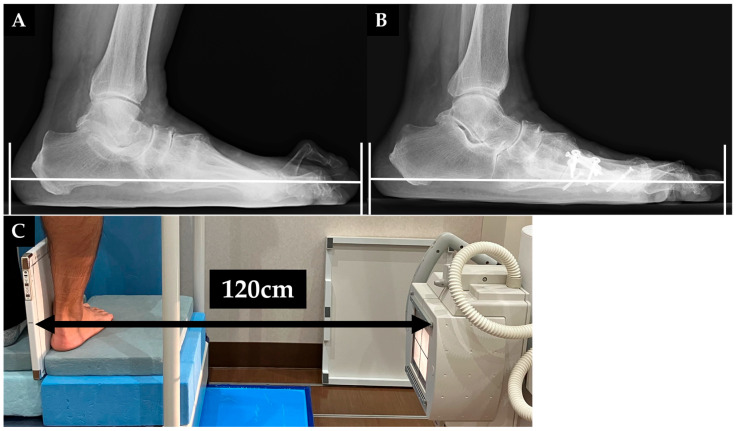
Preoperative (**A**) and postoperative (**B**) measurements of radiologically determined foot length on lateral weightbearing radiographs. Radiologically determined foot length was defined as the distance between the shadow of the heel to the tip of toe (white line). The patients stood on the table; then, their feet were positioned in a natural standing posture, ensuring that the toes faced directly forward. The detector was placed vertically against the inner side of the foot. The focal spot-to-film distance was set to 120 cm. The incidence angle was 0 degrees (**C**).

**Figure 2:**
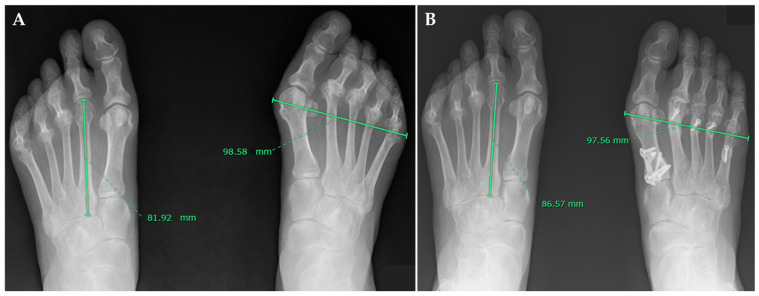
Preoperative (**A**) and postoperative (**B**) measurements of radiologically determined foot width on anteroposterior weightbearing radiographs. Radiologically determined foot width was defined as the maximal distance between the most medial and lateral shadow of the foot. By measuring the ratio of the contralateral second or fifth metatarsal bone length between the pre- and postoperative radiographs, we were able to compensate for all small changes in length.

Radiologically determined foot length was assessed by measuring the maximal distance between the heel and the tip of toe (Figure 1A,B). The patients stood on the table; then, their feet were positioned in a natural standing posture, ensuring that the toes faced directly forward. The detector was placed vertically against the inner side of the foot. The focal spot-to-film distance was set to 120 cm. The incidence angle was 0 degrees (Figure 1C). Radiologically determined foot width was assessed by measuring the maximum distance between the most medial and lateral soft tissue outlines at the level of the first metatarsal head in a same method to previous reports on forefoot width [17]. The weightbearing dorsoplantar views of the foot were examined. Radiographs were obtained with the subjects in a standing position with the knees in full extension. A specific detector was placed under the feet to avoid projection and magnification artifacts (Figure S1A,B). Irradiation was then performed from a distance of 100 cm and tilted forward by 30° from the vertical [18]. To maximize measurement accuracy, we calibrated the length measurements (soft tissue maximal width) according to the contralateral lesser metatarsal bone length, on which additional surgery had not been performed during the follow-up period and which was radiographed simultaneously. By measuring the length ratio of the contralateral second metatarsal bone or the fifth metatarsal bone between the pre- and postoperative radiographs, we could compensate for small variations in length caused by possible different angles or distances in which the radiographs were taken [17,19] (Figure 2A,B). Pre- and postoperative radiographic assessments included measuring the hallux valgus angle (HVA), intermetatarsal angle between the first and second metatarsals (M1M2A), and intermetatarsal angle between the first and fifth metatarsals (M1M5A) using Miller’s method on anteroposterior weightbearing radiographs [20]. The CP angle was the angle formed by the line drawn from the plantar surface of the calcaneus to the inferior border of the calcaneocuboid joint, compared to the horizontal line [21]. An author examined the radiographic assessments (M.H.). We evaluated the correlation between changes in radiographic parameters and variations in radiologically determined foot length and width.

### 2.2. Operative Technique and Postoperative Protocol

In the joint-preservation group, modified Mann method was performed as previously described [15,16]. Following osteotomy and correction, internal fixation was performed using a plate (DARCO^®^, Wright Medical Technology, Memphis, TN, USA) in 33 feet. For the lesser toe, we performed the modified SOO procedure and manipulation of the proximal interphalangeal (PIP) joint to correct lessor toe deformities; then, the metatarsal bone was fixed with a screw [15,22,23]. Cannulated cancellous screws (DARTFIRE^®^, Wright Medical Technology) were used to fix the osteotomy site in 30 cases while cortex screws (Modular Hand System, DePuy Synthes) were used in 3 cases following a previously described procedure [15,22]. Extensor plication and dermodesis were performed on the PIP joints of 10 toes from 7 feet. A Z-lengthening procedure was performed on the extensor digitorum longus (EDL) tendon and the extensor digitorum brevis (EDB) tendon was released in 82 toes from 27 feet.

In the joint-sacrifice group, arthrodesis of the first metatarsophalangeal joint was performed as previously described [16,23]. Two kinds of cannulated cancellous screw (DARTFIRE^®^ [Wright Medical Technology, Memphis, TN, USA] and ACE screw [Zimmer Biomet, Warsaw, IN, USA]), and two types of headless compression screws were used: Acutrak 2 mini screw [Acumed, Beaverton, OR, USA] and HCS [Depuy Synthes, Solothurn, Switzerland]). Double-thread headless screws (DTJ screw, MEIRA©, Nagoya, Japan), and headless compression screws (Acutrak 2 -mini screw, Acumed, Beaverton, OR, USA) were also used. The number of cases for each type is as follows: 26, 4, 4, 1, and 2, respectively. The first MTP joint was fused using a locking plate (MPJ Fusion plate; Wright Medical Technology) and a headless compression screw (Acutrak 2 mini screw, Acumed) in two feet. For the lesser toe, we performed the modified SOO procedure using cannulated cancellous screws (DARTFIRE^®^, Wright Medical Technology) in 21 cases and cortex screws (Modular Hand System, DePuy Synthes) in 2 cases following a previously described procedure [15,22,23]. Resection arthroplasty was performed as previously described [15,16]. The PIP joint was manipulated in all feet. Extensor plication and dermodesis were performed on the PIP joints of ten toes from seven feet. Extensor release and joint resection at the PIP joint were performed in one toe. A Z-lengthening procedure was performed on the EDL tendon and the EDB tendon was released in 87 toes from 28 feet. In both groups, the surgical operations were performed by 4 surgeons. Postoperative management was performed for all patients based on our previous protocol [15,22,23].

### 2.3. Statistical Analysis

Statistical analyses were performed using Easy R [24]. To compare the outcomes before and after surgery, the Wilcoxon signed-rank test was used. When testing the correlation between changes in radiographic parameters with variations in radiologically determined foot length and width, the Spearman rank correlation coefficient was used. Fisher’s exact test was used to compare the number of variations in radiologically determined foot length and width between joint-preservation and joint-sacrifice groups. A *p* value of < 0.05 was considered statistically significant.

## 3. Results

Radiologically determined foot width changed significantly from 101.7 mm to 97.3 mm (*p* < 0.001), while there was no significant difference between pre- and postoperative radiologically determined foot length. In total, 36 feet showed extended radiologically determined foot length, with the foot length increasing by a maximum of 19 mm and decreasing by 17 mm postoperatively, while 60 feet showed decreased foot width, with the foot width increasing by a maximum of 4.3 mm and decreasing by 12 mm postoperatively. Pre- and postoperative radiographic parameters showed that the HVA, M1M2A, M1M5A, and CPA were significantly reduced from 50.1° ± 18.1° to 20.6° ± 9.6° (*p* < 0.001), 13.1° ± 4.7° to 9.7° ± 4.3° (*p* = 0.001), 33.5° ± 7.9° to 27.8° ± 7.9° (*p* < 0.001), and 17.0° ± 7.8° to 16.0° ± 7.4° (*p* < 0.001), respectively (Table 2).

A significant negative correlation was found between the variation in radiologically determined foot length and changes in HVA (r = −0.28, *p* = 0.016) and M1M5A (r = −0.23, *p* = 0.048), while no significant correlation was found between the variation in radiologically determined foot length and changes in M1M2 and CPA. A significant positive correlation was found between the variation in radiologically determined foot width and changes in HVA (r = 0.34, *p* = 0.003), M1M2A (r = 0.53, *p* < 0.001), and M1M5A (r = 0.45, *p* < 0.001), while no significant correlation was found between the variation in radiologically determined foot length and change in CPA (Table 3). There was no significant correlation between variations in radiologically determined foot length and width and the following characteristic parameters: age, disease duration, DAS28-CRP, the use of MTX, the use of PSL, the use of bDMARDs, and HAQ-DI score. In total, nine feet (12.5%) had increased by more than 5% in length and 28 feet (38.9%) had decreased by more than 5% in width. There were no significant differences between operative procedures regarding variation in radiologically determined foot length and width (Table 4 and Table 5).

## 4. Discussion

In the current study, we demonstrated for the first time that radiographic foot width decreased significantly after joint-preservation and joint-sacrifice procedures for rheumatoid forefoot deformities. Both procedures for forefoot deformities also maintained radiographic foot length on weightbearing radiographs. These results suggested that our procedures for rheumatoid forefoot deformities could reduce radiographic foot width while maintaining foot length.

Regarding research on radiographic foot width after surgery for HV, a recent study found that a modified Lapidus procedure decreased radiographic foot width by 6.8% with weightbearing for at least five months after surgery [5]. In addition, a modified Lapidus procedure decreased radiographic foot width by 5.85% with weightbearing for at least six months after surgery [25]. Although chevron osteotomy decreased radiographic foot width by 7.1% with weightbearing for at least 12 months after surgery [26], minimally invasive chevron osteotomy with Akin bunionectomy decreased radiographic foot width by 2% with weightbearing for at least three months after surgery [27]. Scarf midshaft osteotomy reduced radiographic foot width by 2% with weightbearing for at least six months after surgery [17]. These studies have conflicting results, as they evaluated different osteotomy sites and used different landmarks on the first ray to calculate the medial limits. In this study, radiographic foot width decreased by 4.2% with weightbearing for at least six months after surgery, which is consistent with the findings of previous studies that corrected HV deformities using the Lapidus, Chevron, and Scarf procedures. In addition, 38.9% of patients had a decrease in radiographic foot width of more than 5%, similar to a previous study in which 45.1% of patients exhibited a decrease in radiographic foot width of more than 5% [17].

Our radiographic assessments demonstrated that HVA, M1M2A, and M1M5A significantly improved after surgery in both joint-preservation and joint-sacrifice procedures. They also demonstrated that changes in M1M2A and M1M5A were moderately correlated with the variation in radiologically determined foot width, while there was a low correlation between changes in the HVA and radiologically determined foot width. Previous studies reported that changes in HVA and IMA were moderately correlated with variations in radiographic foot width after Chevron Akin and Lapidus procedures for HV [27,28,29]. In patients with RA, the average CPA decreased significantly at the latest follow-up when compared to preoperative values [21]. In the current study, the average CPA also decreased significantly at the final follow-up compared to preoperative values. However, there were no significant correlations between changes in CPA and variations in radiologically determined foot length and width. These results suggest that adequate correction of M1M2 and M1M5 may be an effective way of achieving a narrow foot.

The ability to wear preferred footwear was reported to be more important to QOL following HV surgery than either hallux valgus angle (HVA) or intermetatarsal angle (IMA) correction [8]. In addition, radiographic foot width reduction has been reported to be a desirable cosmetic and functional outcome for patients with HV [5]. The current study found a significant correlation between improvements in each angle and radiographic foot width. This suggests that joint arthroplasty for forefoot deformity could contribute to improving radiographic foot width in patients with RA.

Although no study has yet examined the effect of joint arthroplasty on the foot length and width in patients with rheumatoid forefoot deformities, a longitudinal study of foot anthropometry in RA patients demonstrated that those with high disease activity experienced a greater decrease in foot length and a greater increase in foot width due to the progression of HV, lesser toe deformities, and spread feet [2,4]. Combined treatment with MTX and bDMARDs is effective in controlling disease activity and modifying foot structure [4]. In the current study, no significant difference was detected between the patients with and without bDMARDs in terms of variations in radiologically determined foot length and width. In addition, there was no significant correlation between variation in radiologically determined foot length and width and DAS28-CRP. This may be because we often indicated the surgery for RA patients whose disease activity was well-controlled by medications. This study found a significant negative correlation between the variation in radiologically determined foot length and changes in HVA and M1M5A. Although the metatarsal length was shortened by surgical osteotomy and MTP joint fusion, these results suggest that improvements in HV and spread feet may contribute to maintaining radiographic foot length. In addition, there were no significant differences between the joint-preservation and sacrifice procedures regarding variation in radiologically determined foot length and width. A study reported that patients with RA hope to return to normal in terms of physical function, disease control, social participation, and mental function [30]. These results suggest that both procedures could improve radiographic foot width and maintain the foot length, which may contribute to improving foot appearance and patients’ ability to wear a range of shoes.

The present study has several limitations. First, the indications for joint-preserving and joint-sacrificing procedures were not randomly assigned. The surgical indication depended on the patients’ age, disease activity control, and level of ADL, HVA, and MTP joint destruction. Second, our sample size was small and the follow-up period was relatively short. Third, although we investigated radiologically determined foot length and width, podometric measurements rather than radiographic foot length and width could have provided pertinent information to this investigation. Fourth, our method for assessing radiologic foot length and width has not been validated. Although this measurement system using the length ratio of the second metatarsal bone or the fifth metatarsal bone was reported to be excellent interobserver reliability for pre- and postoperative radiographic foot width for HV (27), we could not use the same measurement system because surgical procedure for rheumatoid forefoot deformity was often performed for all metatarsal bones. Alternatively, to calibrate the length measurements (soft tissue maximal width) we used the contralateral lesser metatarsal bone length, on which additional surgery had not been performed during the follow-up period and which was radiographed simultaneously.

This study lacks the segmental measurements required to support the hypothesis that toe realignment compensates for the osteotomy and power analysis. The impact of foot width reduction due to joint arthroplasty on the functional capacity and ability to wear a range of shoes has not yet been examined. Further research should focus on how changes in foot width after surgery affect the patient’s ability to wear their preferred footwear and their satisfaction. Additionally, we should determine the minimal clinically important difference for this parameter. However, while we acknowledge the limitations of our investigation, the strength of the current study is that it is the first to report the effects of joint arthroplasty on variations in foot length and width in patients with rheumatoid forefoot deformities. Further prospective and comparative studies with larger populations are required to evaluate the effects of forefoot width changes on clinical and functional outcomes.

## 5. Conclusions

In conclusion, the surgical procedure for rheumatoid forefoot deformity improved radiographic parameters and reduced radiographic foot width while maintaining foot length. Furthermore, this study demonstrated that the radiographic parameters of the HV, particularly the IMA, potentially affect forefoot width.

## Figures and Tables

**Table 1:** Patients’ demographic and clinical parameters (N = 72 feet in 53 patients).

Characteristic			
Age (years)	66.4	±	8.5
Gender (Male : Female)	3	:	50
Disease duration (years)	23.7	±	9.3
Follow-up period (months)	17.5	±	10.4
MTX	36	(67.9)	
PSL	15	(28.3)	
bDMARDs	22	(41.5)	
DAS28-CRP	2.5	±	0.8
HAQ-DI	1.0	±	0.7

All values are expressed as mean ± standard deviation or *n* (%).

**Table 2:** Pre- and postoperative radiographic measures (N = 72 feet in 53 patients).

Variable	Preoperative			Postoperative			*p* Value	
Foot length (mm)	227.2	±	12.3	227.8	±	14.2		0.877
Foot width (mm)	101.7	±	7.4	97.3	±	6.5	<	0.001 *
HVA (°)	50.1	±	18.1	20.6	±	9.6	<	0.001 *
M1M2A (°)	13.1	±	4.7	9.7	±	4.3	<	0.001 *
M1M5A (°)	33.5	±	7.9	27.8	±	7.9	<	0.001 *
CPA (°)	17.0	±	7.8	16.0	±	7.4	<	0.001 *

All values are expressed as mean ± standard deviation. * Statistically significant (*p* < 0.05).

**Table 3:** Correlation between changes in angular parameters and variations in radiologically determined foot length and width (N = 72 feet in 53 patients).

Change in Angular Measurement	Δ-Foot Length (mm)		Δ-Foot Width (mm)	
Δ-HVA (°)	−	0.28 *		0.34 *
Δ-M1M2A (°)		0.04		0.53 *
Δ-M1M5A (°)	−	0.23 *		0.45 *
Δ-CPA (°)	−	0.14	−	0.08

* Statistically significant (*p* < 0.05).

**Table 4:** Change in radiologically determined foot length in joint-preservation and joint-sacrifice group (N = 72 feet in 53 patients).

	Joint-Preservation (*n* = 33)			Joint-Sacrifice (*n* = 39)		
		Change in Foot Length			Change in Foot Length	
Shortening of foot length, *n*, mm	15	−	6.5	21	−	6.2
Extension of foot length, *n*, mm	18		6.4	18		8.6

All values are expressed as n, mean change in radiologically determined foot length.

**Table 5:** Change in radiologically determined foot width in joint-preservation and joint-sacrifice group (N = 72 feet in 53 patients).

	Joint-p Reservation (*n* = 33)			Joint-Sacrifice (*n* = 39)		
		Change in Foot Width			Change in Foot Width	
Narrowing of foot width, *n*, mm	27	−	5.3	33	−	6.2
Widening of foot width, *n*, mm	6		2.2	6		2.7

All values are expressed as *n*, mean change in radiologically determined foot width.

## Data Availability

The data of this study are included within the article and available from the corresponding author on reasonable request.

## Supplementary Materials

The following supporting information can be downloaded at: https://www.mdpi.com/article/10.3390/jcm15051877/s1, Figure S1: Method of weightbearing radiography.

## Abbreviations

The following abbreviations are used in this manuscript:

MTX	methotrexate
PSL	prednisolone
bDMARDs	biologic disease-modifying antirheumatic drugs
DAS28-CRP	disease activity score 28 c-reactive protein
HAQ-DI	health assessment questionnaire-disability index
HVA	hallux valgus angle
M1M2A	intermetatarsal angle of the first and second metatarsals
M1M5A	intermetatarsal angle of the first and fifth metatarsals
CPA	calcaneal pitch angle
