# Supplementary material for: Effect of Surgical Procedures for Rheumatoid Forefoot Deformities on Radiographic Foot Length and Width Variations
Source: J Clin Med. 2026 Feb 28;15(5):1877. doi: 10.3390/jcm15051877 (PMC12986326; doi:10.3390/jcm15051877)
Supplement: Supplementary file 1 [file jcm-15-01877-s001.zip › jcm-4143248-supplementary.pdf]

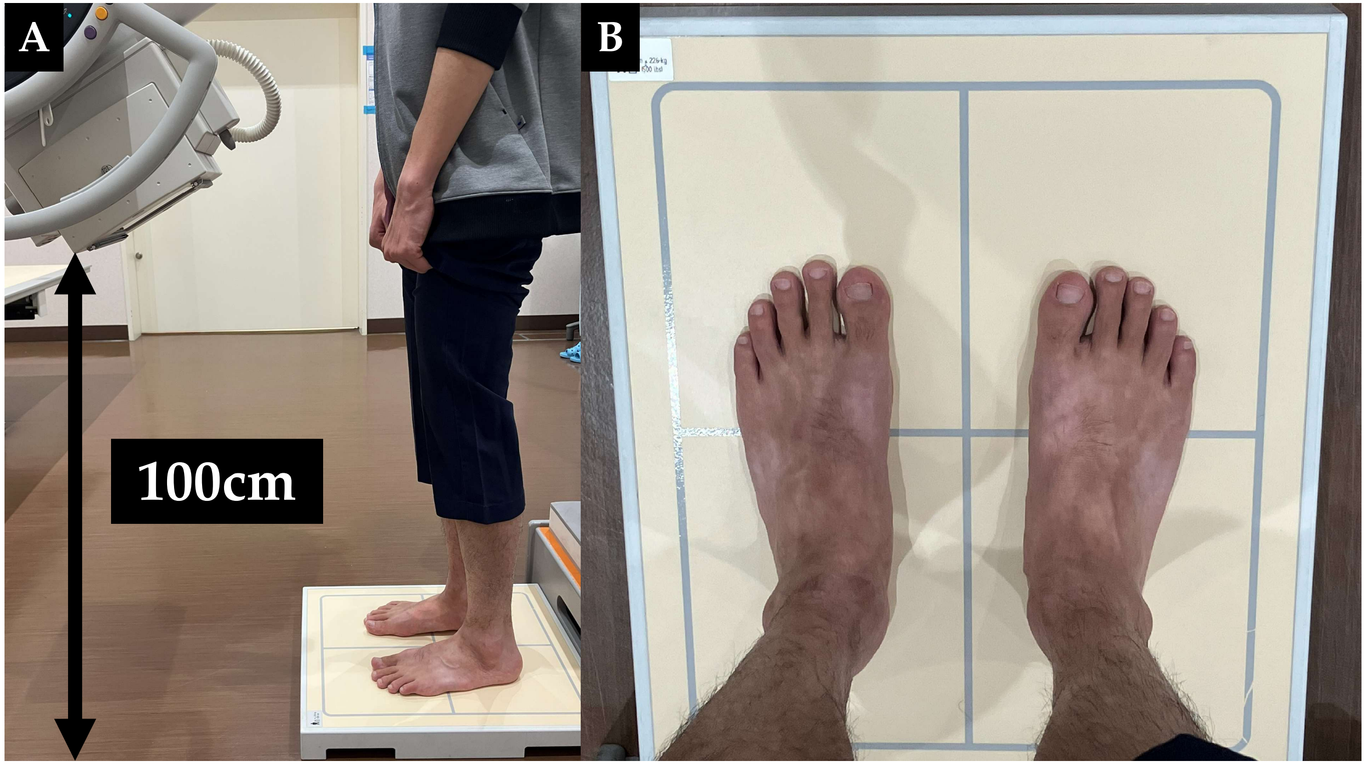

Figure S1 .

A specific detector was placed under the feet to avoid projection and magnification artifacts. Irradiation was then performed from a distance of 100 cm and tilted forward by 30° from the vertical (A, B).
